# Reducing the risk of heart disease among Indian Australians: knowledge, attitudes, and beliefs regarding food practices – a focus group study

**DOI:** 10.3402/fnr.v59.25770

**Published:** 2015-06-05

**Authors:** Ritin Fernandez, John X. Rolley, Rohan Rajaratnam, Bronwyn Everett, Patricia M. Davidson

**Affiliations:** 1 School of Nursing, University of Wollongong, Wollongong, NSW, Australia; 2 Centre for Research in Nursing and Health, St George Hospital, Kogarah, NSW, Australia; 3 School of Nursing and Midwifery, Strategic Research Centre for Quality and Patient Safety, Deakin University, Melbourne, Victoria, Australia; 4 South Western Sydney Local Health District, Liverpool, NSW, Australia; 5 University of Western Sydney, Parramatta, NSW, Australia; 6 Centre for Applied Nursing Research, Ingham Institute, South Western Sydney Local Health District, Liverpool, NSW, Australia; 7 School of Nursing, Johns Hopkins University, Baltimore, MD, USA; 8 Centre for Cardiovascular and Chronic Care, Ultimo, NSW, Australia

**Keywords:** Asian Indians, South Asian, diet, heart disease, food practices, attitudes, knowledge

## Abstract

**Background:**

Australia has a growing number of Asian Indian immigrants. Unfortunately, this population has an increased risk for coronary heart disease (CHD). Dietary adherence is an important strategy in reducing risk for CHD. This study aimed to gain greater understanding of the knowledge, attitudes and beliefs relating to food practices in Asian Indian Australians.

**Methods:**

Two focus groups with six participants in each were recruited using a convenience sampling technique. Verbatim transcriptions were made and thematic content analysis undertaken.

**Results:**

Four main themes that emerged from the data included: migration as a pervasive factor for diet and health; importance of food in maintaining the social fabric; knowledge and understanding of health and diet; and elements of effective interventions.

**Discussion:**

Diet is a complex constructed factor in how people express themselves individually, in families and communities. There are many interconnected factors influencing diet choice that goes beyond culture and religion to include migration and acculturation.

**Conclusions:**

Food and associated behaviors are an important aspect of the social fabric. Entrenched and inherent knowledge, attitudes, beliefs and traditions frame individuals’ point of reference around food and recommendations for an optimal diet.

Despite advances in medical technology and pharmaceutical therapy, coronary heart disease (CHD) remains the leading cause of mortality and morbidity worldwide (1). Asian Indians, in particular, have been found to have higher levels of risk for CHD both in country of origin, as well as those who make up the diaspora. In addition, Asian Indians experience a first myocardial infarction at a much younger age (2) and mortality because CHD is five- to tenfold higher in those aged under 40 years (3).

Adherence to dietary best practice recommendations is, among other critical factors, essential for primary and secondary prevention of CHD (4). Recommendations involve consumption of a varied diet high in wholegrain cereals, fruit and vegetables, foods low in salt, and limited consumption of saturated fats, sugar, and foods containing added sugars. In addition, the Australian Guide to Healthy Eating provides people with a visual representation to assist the selection of healthy foods (5). Measuring diet empirically, however, is difficult particularly in terms of accurately reporting intake in relation to overeating and disease (6).

## Indians and diet

Dietary customs and habits among Asian Indians are varied depending on their region of origin in India, cultural, and religious beliefs (7). The traditional Indian diet is carbohydrate dense and lacks high-quality protein and antioxidants (8). In addition, the Indian diet comprises large amounts of added sugars (9, 10), large portions (11), and late dinners.

Dietary patterns associated with immigration and acculturation may contribute to a higher risk of heart disease among Asian Indians (12). Asian Indians make up a steadily growing diaspora estimated to be over 20 million worldwide (13). In 2011, Indian-born Australians were the second largest overseas-born Asian group in Australia, and the fourth largest overseas-born group overall (14). In addition to permanent migrants, Australia has a large temporary Asian Indian population in the form of tertiary students coming to study in Australia. Given the evidence of increased CHD among Asian Indians particularly at a younger age, health professionals, and nurses in particular, can provide dietary education to reduce the risk of heart disease in this population.

To date, little research has been done on the complex interplay of psycho-socio-biological factors on food practices in Asian Indians. In particular, no research has been done on the migrant population of Asian Indian Australians. Therefore, understanding the knowledge, attitudes, and beliefs influencing food practices is vital in order to develop culturally appropriate interventions for diet-related behavior change. Rather than measuring these constructs quantitatively, a more in-depth qualitative approach may provide greater insights into the ways these factors interact.

This paper reports and discusses the findings of a qualitative study into knowledge, attitudes, and beliefs relating to food practices and strategies for the prevention of heart disease among Asian Indian Australians.

## Methods

### Participants

A convenience sample of migrant Asian Indian Australians who took part in a larger risk factor profile study was recruited to participate in a focus group. Focus groups are particularly useful for exploring complex issues about which little is known. However, it is the dynamic of social interaction among the participants of focus groups that helps elicit rich data through the mutual support members’ experience. That support aids deep discussion and therefore rich findings (15, 16).

Using a convenience sample, participants who agreed to take a survey associated with the larger study (17) were invited to attend a focus group. Inclusion criteria included adults who identified themselves as Asian Indians who were either migrants to Australia, or born to Asian Indian migrants living in Australia. The sample consisted of Asian Indian Australian adults who were capable of conversing freely in English. This was important as participants came from varying Asian Indian language groups. To provide equal access, the English language was chosen. Furthermore, a high level of English language skills is known to exist in the Asian Indian population (18). The focus groups were conducted at the university after consultation with participants to ensure that the venue was suitable to them.

### Data collection

Community leaders of local cultural associations and members of the Indian Medical Associations were contacted from a list provided by the Consul General of India to participate in a larger study titled ‘Heart Health and Well being among Asian Indians Living in Australia’ – a study undertaken to develop and implement an evidence-based intervention to reduce the risk of heart disease among Asian Indians. Participants were contacted by email and invited to participate in this study if they had previously provided the researchers their contact details as part of the larger study, and indicated an interest in participating in a focus group. A letter providing details of what was required during the focus group session was sent out along with a form to establish the most convenient time and location for the focus group. An information sheet was also sent out outlining the key areas of discussion regarding the Asian Indian community relating to practices about wellness, heart health, and preventive health, the various health and community resources available, and what they perceived to be the components of an effective program for reducing the risk for heart disease. Written consent was obtained from each participant prior to conducting the focus groups.

### Measures

Open-ended questions were developed based on the risk factor data obtained from a substudy of the *Heart Health and Well Being among Asian Indians Living in Australia Project*. These questions related to their perception of their food practices, its impact on heart health, and strategies that would enable people in their community to eat healthy. Focus groups were conducted using a dual moderation approach as difficulties have been noted in single-moderator studies where the moderator is required to ask questions as well as keeps field notes (19). During the focus group sessions, feedback methods were used by the moderators to reflect back to the participants pertinent issues raised during the dialog. Each focus group lasted for 90 min, and data saturation was reached following the second focus group. Each focus group was digitally audio-recorded and transcribed verbatim to allow independent analysis by the research team. Field notes were compiled by each facilitator for inclusion in the analysis. Following the focus group sessions, the moderators met to debrief, note any common themes, and discuss the field notes gathered during the session. These notes informed the data analysis process.

### Analysis

Following transcription, data were analyzed for emergent themes and subthemes. Three researchers independently analyzed the data and later discussed their results before arriving at a consensus of the essential themes and subthemes (20). Exemplars were selected to illustrate emergent themes and subthemes.

Ethical approval for the study was obtained from the University of Western Sydney Human Research Ethics Committee (approval no.: H8403).

## Results

Two focus groups were held with a combined sample of 12 participants (Group 1: 6 and Group 2: 6). The majority of the participants were male (n=9). Participants had migrated from South, North, and Northeast India thus representing several subcultures of the region.

Participants were aged between 35 and 70 years, all had completed a Bachelor's degree and only one was born in Australia. Two participants were retired but were actively working in their community groups. Participants in the study included a general practitioner, accountants, dietitians and financial advisors. Their length of stay in Australia ranged from 5 to 40 years.

The main themes that emerged from the focus groups included (1) migration as a pervasive factor for diet and health; (2) the importance of food in maintaining vital social fabric; (3) knowledge and understanding of health and diet; and (4) preventing heart disease and improving health.

### Migration as a pervasive factor for diet and health

Indian Australians identified the challenges of migration as negatively influencing dietary practices and health. Subsumed within this theme were challenges relating to stress and under-employment, loss of the extended family, and financial pressures.

#### Stress and under-employment

Migration as a source of considerable stress was discussed by the majority of participants. The stress associated with migration, particularly for skilled migrants, was substantial. Participants discussed the challenge migration presented in terms of affordability for living in the new environment. One aspect was under-employment of professionally trained migrants. This under-employment had a perceived impact on the health of the family, particularly the dyad as both husband and wife needed to work. As one participant expressed:

[P]: … when we came here the whole thing changed, the whole place changed … the women had to look for a job, the children are neglected, the food were prepared in haste.

The introduction of fast food was implicated in this process and, therefore, the westernization of their diet leading to a perceived reduction in health. One participant stated:

[M2]: … when I was working … lunch becomes a fast food type of thing … you get used to this … if it's pizza or whatever you want to eat you see, or McDonalds.

#### Access to low-cost low-nutrient foods

Migration also added financial pressures to new immigrants leading to increased risk factors for heart disease. In particular, unhealthier food choices being cheaper than more nutritious foods were demonstrated in the discussions. Examples included ice cream, pizza, and beer all being cheaper to consume. As one participant described his transformation upon migration:

[J]: … basically I never had ice cream when I was in India. I came here as a student and I found ice cream was the cheapest to eat … You know I'm not joking, when I came to Australia I was only 75 kg. In three years I was 120 kg and now I'm 100 kg.

Another participant echoed the above comments elucidating on the link between diet and exercise:

[P]: … when I came here I was 128 pounds, now I'm 228 [pounds]. You didn't take out what you put in, and you didn't walk too long, we use the cars. Back home we used to walk to work. The drinks [beer] were so cheap when I came here … buy a carton of beer for about $5.99 … we used to drink a carton a week.

#### Loss of the extended family

The loss of the extended family as a major social support was identified. The notion of family had to be redefined by including friends as surrogates for that loss of extended family. As one participant stated:

[J]: … Back home in India … my grandmother was the one who took care of me. So I was getting proper food and not like you know fast food kind of thing.

Participants identified differences between traditional dietary practice and post-migration practice. For example, the number of times a person eats per day has changed. Prior to immigration, it was common to eat several small meals per day

[P]: We have the habit of eating five meals a day, when we came here we just eat three meals because we don't even have time.

### Importance of food in maintaining vital social fabric

Participants discussed the role food played within the traditional contexts of family and community. From the patterns of meals and communal eating to maintaining social cohesion, food was seen as integral to Asian Indian culture. As one participant discussed:

[M]: … almost every weekend we socialize. So when we invite somebody, we have all those items [food] and we eat as much as possible.

Beliefs around the importance of types of foods during social events were also expressed including ‘sweets’ as a culmination to a meal. Two participants illustrated this well:

[L]: … some have a sweet tongue … without … sweets they are not satisfied.

Responding to this comment, another participant stated:

[K]: The meal isn't over.

### Knowledge and understanding of health and diet

When asked of their knowledge of the connection between diabetes and heart disease, some participants were not aware of the link. One participant made the following comment:

[J]: … My family, there's nobody with heart disease. But with diabetes yes. But to be frank with you I still eat sweets and I don't think that will be a problem in my life.

Other misconceptions about health, diet, and heart disease were also expressed, in particular, the issues related to risk factors for heart disease. Being overweight was not necessarily seen as a health-negative issue. Participants discussed the cultural aspects of this notion. When asked how the community in general regarded being overweight, one participant who was a health professional stated:

[L]: I think they disregard it probably …. They know, ‘I am overweight’, but still when they see a piece of sweets they forget about it [the weight].

Fatalism regarding health and health outcomes was noted by participants. This was expressed more in terms of comparing the apparent irony of a person of advanced age with multiple risk factors yet appearing well.

[J]: … people tend to compare instances, for example say so and so … had no problems … still he passed away at 50. [another] person was having all sorts of problems, overweight, diabetic and what not, still he is 90 he is still going strong …

### Preventing heart disease and improving health

Participants had much to say about aspects of interventions that may improve health and dietary outcomes. These centered on the family, community, and the use of media.

#### The family as a driver for change

The family was singled out specifically as an important unit for primary and secondary prevention strategies. In particular, the woman's role within the household was emphasized as she was considered the primary preparer of food:

[M]: … in our community, food is normally prepared by the lady at home … so awareness of those [issues] to the women is more important … for instance my wife, she decides what she should cook and how she could cook.

Women were also the ones considered the most knowledgeable concerning dietary issues. One participant commented:

[M2]: … my wife is more conscious about health issues than I am …

#### Community empowerment

The Asian Indian Australian communities were also identified as important contexts for heart disease prevention interventions. One participant stated clearly:

[M2] … awareness and education within the community [Asian Indian Australian] is something which we need to do.

Discussion included the use of cultural fairs, religious settings, such as Hindu and Sikh temples, and community settings such as grocery stores and restaurants. By way of example, the following participant encouraged the use of cultural fairs emphasizing the large numbers of community that attend:

[M]: … a good number attend. I mean you cannot cover all the community … but majority … around 25,000 people … that's a big number that you can get at one place.

Religious settings featured as alternative contexts for interventions. The deeply integrated nature of religion with everyday life was emphasized. As one participant expressed:

[M2]: … the number of temples which have come up in Sydney since I came here … they [the communities] may go for social events and other things, but here the religious thing is a very important thing.

Although the majority of the participants were Hindus, other religions including Sikhism, Buddhism, and Christianity were also discussed. The emphasis was on the role of religious gathering as a context for potential intervention.

Other settings included shopping centers, in particular, culturally-specific shopping areas frequented by Asian Indian Australians.

[M2]: Maybe community grocery shops you see, not the supermarkets so much because they may not provide that type of thing [dietary intervention].

Like other settings, the timing of delivering such an intervention in the community grocery context was considered important.

[J]: Especially the weekends, because on weekends is when much of [the] people go there [Asian groceries].

#### Media as a change agent

Media was the third identified area for focus in developing and delivering a dietary-related intervention with particular emphasis on television and radio.

[M2]: You've got SBS radio now, a Hindi program … they've started a new service … disability which is again an educational awareness thing.

Print media was also discussed. The many Asian Indian languages and dialects were discussed. However, the provision of health information in the most common languages used by the Asian Indian Australian community was seen as important along with the frustration that the government bodies have little understanding of the complex linguistic needs of the Asian Indian communities.

[M2]: Within our community we've got about 12 languages or even more … 18 languages. Unless you have … language specific booklets, information ones, they won't understand … for instance, we persuaded the health department to produce handbooks in Tamil language, which is again a major language. They thought only Hindi was a major language.

Aging members of the community were singled out as particularly in need of linguistically-diverse material.

[M2]: Older people need it … it's an aging community you see.

Language was also considered important when engaging health professionals. The example of a dietician was mentioned.

[L]: … in my medical centre we've got a very good dietician, where we send the majority of our people … They speak the same language too, so it's very easy for them.

Emphasis was also placed on the temporary migrants including students and the relatives that visit regularly. The following expresses the concerns of the participants well:

[L]: Even more … there are a lot of students [that] have come here, have got permanent residency, and their parents are coming regularly … most of them are visitors, but they come every year ‘cause they've got 10 year permit. They require this type of help in the local language.

## Discussion

This paper presents the findings from a focus group study of Asian Indian Australians and their perceptions of heart disease and diet. The findings from this study provide insight into the challenges of achieving improved cardiovascular health outcomes amidst misconceptions regarding what constitutes a healthy diet. Migration as a substantial catalyst for diet change and subsequent impacts on cardiovascular health is a key finding of this study. In addition, while Asian Indians have similar anthropometric characteristics, cultural, linguistic, and religious attributes remain quite heterogeneous (21) and have a profound and wide-ranging influence on perceptions of health, heart disease, and dietary practice.

An important insight from this study involves how culture forms a vital factor in determining dietary behaviors, as well as how its potential disruption through migration and subsequent acculturative stress can adversely impact on cardiovascular health. This finding is congruent with that reported in other literature on migrant health (22, 23). Asian Indians have their own culturally-based diets and dietary habits comprising mainly of carbohydrate dense foods (24). Biculturalization due to migration could result in consumption of both Indian and Australian food (25) and not replaced with each other. For example, rice and roti continue to be consumed as the main meal, and pizza and burgers as snacks resulting in an even denser carbohydrate diet. These dietary behaviors place the already at-risk Asian Indian population at an even higher risk of cardiovascular disease.

Under-employment and changes to the patterns of how income are brought into the family unit add to the challenges to adaptation to a new environment and consequently on health. In this study, participants expressed concern about unemployment and under-employment and how it affected affordability for living in a country rated as one of the most expensive in the world (26). In a recent survey conducted in Australia, approximately one-fifth of the skilled migrants were either unemployed or under-employed at 6 months following migration, which supports the findings obtained in this study (27). Similar findings have been reported, in particular, where economic hardship hinders healthy adaptation to the new country leading to acculturative stress and a lower self-reported health status. Costs for fresh foods continue to be higher than so-called ‘fast-food’ or ‘take-away’ food resulting in economically-disadvantaged populations opting for the more affordable yet less healthy ‘fast-food’ options.

Health, itself as a construct, is seen from a perspective diverse from that of the dominant Anglo-Saxon Australian point of view. In particular, obesity was not readily perceived as a health-related issue. This finding is interesting as abdominal obesity is a well-established risk factor for heart disease (28), and specific cutoffs for abdominal obesity in Asian Indians (29) have been developed to initiate early management.

In addition, a sense of fatalism governed the perceptions of health. Fatalism describes a belief system where the individual's locus of control over health behavior is externalized (30). Other studies into Asian Indian populations have reported similar issues (17, 31, 32).

Participants identified the role of the family and community as important factors in developing future interventions. This itself is in keeping with the importance of family and wider social cohesion as a determinant of health itself (33). The role of women as providers of meals within the family was identified specifically. In Indian society, men may cook, however, women are generally responsible for everyday cooking. A number of the participants stated that it was the responsibility of the wife or mother to shop and cook, therefore, undertaking further research in this group is vital. In addition, establishing a gender-sensitive approach to education regarding food selection and meal preparation is warranted.

Community approaches to dietary health promotion including media and places of worship were also emphasized. Given the cohesive nature of the Asian Indian Australian communities, such approaches may prove efficacious. Evidence from the literature (34) supports the use of community-based interventions based on theory, informed and initiated by community members in improving dietary habits of people and sustaining the change. There is, therefore, an urgent need to develop strategies that both respect the unique cultural perspectives on health while engaging in appropriate primary and secondary prevention necessary to ameliorate risk. These strategies to improve dietary behavior should build on the existing beliefs and attitudes to reduce the risk of cardiovascular disease.

The major strength of the study is the recruitment of Asian Indian participants from the different regions of India given that the cultural, linguistic, and religious attributes of Asian Indians are highly diverse. In addition, the age range of the participants was varied, thus providing a broad perspective relating to the knowledge, attitudes, and beliefs relating to food practices. The participants in the study were community leaders who were educated and holding jobs that were appropriate to their qualifications at the time of the focus group, although some reported to have been under-employed or unemployed previously. In addition, two participants were health professionals who provided their views about their community from a health perspective. Therefore, the sample was able to cover a broad range of Asian Indian migration experiences while capturing the common themes expressed by the participants of both groups. As such, the study was able to gain greater insights into the role that food plays in their lives. The use of focus groups has been found to facilitate groups with a common characteristic in discussing complex issues (16). The provision in advance of the key questions that would be asked during the focus group also provided participants the opportunity to reflect on their responses prior to attending the focus group.

Despite the evidence obtained from this study, the limitations inherent in undertaking such a study need to be acknowledged. The small sample comprising primarily men limits the extent of generalizability of the findings. While the focus group is an effective method at uncovering data, the information may not cover the depth of experiences as well as one-on-one interviews. Furthermore, the level of control the interviewer or moderator has over the course of the discussion is less in focus groups.

## Conclusion

Food and associated behaviors are an important aspect of the social fabric. Entrenched and inherent knowledge, attitudes, beliefs, and traditions frame individuals’ point of reference around food and recommendations for an optimal diet. There are many interconnected factors influencing diet choice that go beyond culture and religion to include migration and acculturation. Interventions to improve dietary choices and thereby influence cardiovascular health will require a socially cohesive approach, which includes families and communities and recognize social determinants of health.

## New contribution to the literature

1. Provides insights into the knowledge, attitudes, and beliefs relating to food practices and heart disease in Asian Indian Australians for the first time.
2. Highlights from the participants’ perspective, the impact of migration on dietary choice and health outcomes.
